# A Splice Site Variant of *CDK12* and Breast Cancer in Three Eurasian Populations

**DOI:** 10.3389/fonc.2019.00493

**Published:** 2019-06-14

**Authors:** Natalia V. Bogdanova, Peter Schürmann, Yana Valova, Natalia Dubrowinskaja, Nurzhan Turmanov, Tatyana Yugay, Zura Essimsiitova, Elvira Mingazheva, Darya Prokofyeva, Marina Bermisheva, Elza Khusnutdinova, Thilo Dörk

**Affiliations:** ^1^Gynaecology Research Unit, Hannover Medical School, Hanover, Germany; ^2^Radiation Oncology Research Unit, Hannover Medical School, Hanover, Germany; ^3^Department of Genetics and Fundamental Medicine, Bashkir State University, Ufa, Russia; ^4^Department of Clinical Immunology, Hannover Medical School, Hanover, Germany; ^5^Oncology Clinic of Almaty, Almaty, Kazakhstan; ^6^Department of Biology and Biotechnology, Kazakh State National University of Al-Farabi, Almaty, Kazakhstan; ^7^Institute of Biochemistry and Genetics, Ufa Federal Research Centre of the Russian Academy of Sciences, Ufa, Russia

**Keywords:** breast carcinoma, genetic susceptibility, DNA double-strand break repair, chromosome breakage syndrome, founder mutation

## Abstract

CDK12 is a member of the cyclin-dependent kinase family that acts as regulator of DNA damage response gene expression. A c.1047-2A>G splice site variant of the *CDK12* gene was recently reported to strongly associate with hereditary breast and ovarian cancer in patients of Tatar ethnic origin. To gain more insight into the potential risk and the population spread of the c.1047-2A>G variant, we have genotyped three breast cancer case-control series of Tatar, Bashkir and Kazakh ethnicity. We identified c.1047-2A>G in 6/155 cases and 12/362 controls of Tatar ancestry, 0/96 cases and 9/189 controls of Bashkir ancestry, and 1/131 cases and 0/154 controls of Kazakh ancestry (Mantel-Haenszel odds ratio 0.72, 95% CI 0.30–1.70, *p* = 0.45). Consistent with the absence of a large effect, bioinformatic analyses predicted that c.1047-2A>G modulates alternative splicing of a NAGNAG sequence rather than constituting a loss-of-function allele, and RT-PCR analyses of c.1047-2A>G heterozygous lymphocytes verified the usage of the predicted alternative acceptor site. Our study confirms a high prevalence of CDK12*c.1047-2A>G in the Tatar and Bashkir population but excludes a role as a clinically actionable high-risk breast cancer mutation.

## Introduction

Familial risk of breast cancer is associated with high- to moderate-penetrance mutations in genes encoding DNA double-strand break sensors and repair proteins, such as *BRCA1, BRCA2, PALB2, ATM, CHEK2*, and others (1, 2). The hitherto known susceptibility genes account for only part of the familial clustering, and remaining cases could thus be explained by mutations in further DNA repair genes acting in the intracellular DNA damage response. Next-generation sequencing, either as gene panel testing or at a genome-wide scale, has already proven to be useful in identifying additional candidate breast cancer susceptibility genes (1, 3–5). As such mutations are generally rare, there is much interest in the investigation of ethnically homogeneous populations where disease-causing mutations can be enriched due to historical founder effects.

A recent report of Brovkina et al. (6) has indicated a significant association of a c.1047-2A>G splice variant in the *CDK12* gene that was specifically found in 8 of 106 breast cancer cases (7.6%) from Tatarstan. Subsequent genotyping identified this mutation in 9/199 breast cancer patients (4.5%) from the Volga district of Tatarstan while it was present in just 1 of the 238 (0.4%) healthy control patients (*p* = 0.007), suggesting an about 10-fold increased risk for breast cancer. *CDK12* encodes a member of the cyclin-dependent serine/threonine kinase family and is an important regulator of homologous recombinational repair (7). Disruption of *CDK12* underlies the tandem duplicator phenotype that is enriched in several cancers including triple-negative breast cancer, ovarian, prostate, endometrial, and liver cancers (8–12). Loss of CDK12 suppresses the expression of homologous recombinational repair genes, probably via polyadenylation, thereby leading to a so-called “BRCAness” phenotype (7). Combination therapy comprising a PD-1 inhibitor and a PARP inhibitor may be an effective approach in patients with CDK12-deficient cancers (13). It is thus important to clarify the prevalence of germline *CDK12* variants that may represent constitutive cancer susceptibility alleles.

In the present study, we aimed to replicate the association of the c.1047-2A>G splice variant in the *CDK12* gene and to investigate its population-specific prevalence in hospital-based breast cancer case-control studies from Bashkortostan and Kazakhstan.

## Patients and Methods

### Patients

We investigated two breast cancer case-control series from Bashkortostan, Russia, and from Kazakhstan. Both series have been previously used for genetic association studies (14–16). The series from Russia consisted of 1,059 breast cancer patients unselected for family history who had been diagnosed during the years 2000–2007 at the oncological center in Ufa (Bashkortostan). Breast cancer patients in this series belonged to different ethnic groups mainly living in the Volga Ural region of Russia, and included Russians, Tatars, Bashkirs, Ukrainians, and patients of other or mixed ancestry. Median age at diagnosis was 51 years (range 25–85 years), and 7% of patients reported a first-degree relative diagnosed with breast cancer. Healthy population controls included 1,069 volunteers from the same geographic regions, with a similar ethnic distribution and age distribution (median age 46 years, range 18–84 years). For the present association study, cases and controls were selected for Tatar or Bashkir ancestry, respectively. Breast cancer patients from other or mixed ethnic subgroups were not included into the *CDK12* genotyping study. We also genotyped 262 unselected ovarian cancer patients from a previously described case series in Bashkortostan (17).

The series from Kazakhstan consisted of 281 breast cancer patients from Russian or Altaic subpopulations (including 131 Kazakhs) and 653 healthy female controls (including 154 known Kazakhs) that had been ascertained at the State Oncology Institute, Almaty, Republic of Kazakhstan. Patients had a median age at diagnosis of 52 years (range 27–91 years) and the healthy controls a median age of 41 years (range 19–73 years). Genotyping for *CDK12* was limited to the subgroup of cases and controls with confirmed Kazakh ethnicity.

Our study was carried out with informed consent of the probands and was approved by local ethical boards at the respective institutions.

### Mutation Analyses

**Figure 1:**
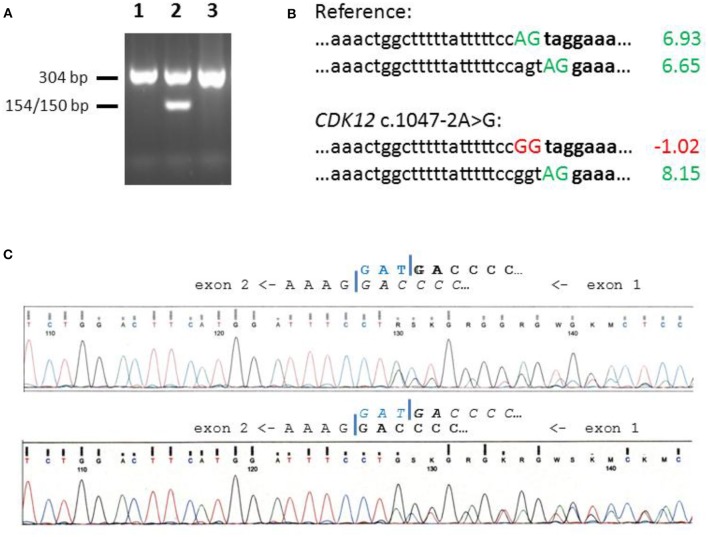
Screening and splice site analysis of *CDK12* c.1047-2A>G. **(A)** Identification of *CDK12* c.1047-2A>G by means of *Msp*I restriction fragment length polymorphism analysis on a 2% agarose gel. Mutation-specific cleavage produces a 154/150 bp band as exemplified in lane 2, with lanes 1 and 3 showing samples with wildtype genotypes. **(B)** Comparative assessment of Maximum Entropy 3′-splice site scores in the wildtype and the mutant context. Splice site scores were obtained from the MaxEntScore site (http://hollywood.mit.edu/burgelab/maxent/Xmaxentscan_scoreseq_acc.html), accessed on Jan 15, 2019. The wildtype sequence harbors two adjacent acceptor splice sites of similar scores (upper two sequences), and the mutant sequence still can make use of the downstream site (bottom two sequences). **(C)** Sanger sequencing of RT-PCR products at the border between *CDK12* exons 1 and 2. Upper panel: wildtype control, Lower panel: *CDK12* c.1047-2A>G heterozygous carrier. Exon borders are indicated by a blue bar, and the main isoform is marked in bold while the minor isoform is indicated in italics. Note that the triplet of the alternative site (TAG, blue) is included in a majority of transcripts represented by the wildtype control sequencing reads, but only in a minority of transcripts from the *CDK12* c.1047-2A>G heterozygous carrier.

Genomic DNA was isolated from peripheral white blood cells by routine phenol-chloroform extraction. A region from the *CDK12* exon/intron boundary that harbors the c.1047-2A>G variant, was amplified using the primers 5′- GGC TGG TTT CTC AGA CTG TC-3′ and 5′-TGA GTT CAG CTC CCA GAC TG−3′. The PCR product of 304 bp was then subjected to restriction enzyme cleavage with *Msp*I, which generates fragments of 154 and 150 bp only in the presence of the c.1047-2A>G variant. The cleavage products were separated through 2% agarose gel electrophoresis supplemented with GelRed Nucleic Acid Gel stain (Biotium, Freemont, USA) and were visualized on a UV transilluminator (Figure 1A).

For studies of alternative splicing, total RNA was isolated from fresh blood samples of one patient heterozygous for *CDK12* c.1047-2A>G and from three patients and one healthy control without this variant, using a standard Trizol guanidinium-phenol extraction protocol. RNA was reverse transcribed using a cDNA synthesis kit (ThermoFisher) with random hexamer primers. RT-PCR was performed with 1 μl cDNA per 20 μl reaction using primers 5′-TCCTGAGCAAGCGGTCTCTG-3′ and 5′-TGAGTTCAGCTCCCAGACTG-3′. Sanger sequencing of RT-PCR products was performed with the reverse primer using BigDye chemistry on a 3130 Genetic Analyser (Applied Biosystems).

### Statistical and Bioinformatic Analyses

The prevalence of the c.1047-2A>G variant was compared in cases and healthy population controls stratified by ethnicity. Odds ratios (OR) per study were calculated from two-by-two tables and statistical significance was assessed with Fisher's exact test (2 df). Mantel-Haenszel odds ratios were then calculated from a fixed-effects meta-analysis of the Tatar, Bashkir and Kazakh case-control studies. An increment of 0.1 was added to allow for zero fields. All analyses were performed using STATA 12.0 (StataCorp LLC., Texas, USA).

For the *in silico* assessment of 3′-splice site strength, the MaxEntScore algorithm was used online to score 23 mers in the mutant and wildtype context (http://hollywood.mit.edu/burgelab/maxent/Xmaxentscan_scoreseq_acc.html). A splice site was considered favorable over another if the score was higher in the Maximum Entropy model. We also made use of Human Splicing Finder as a second algorithm (http://www.umd.be/HSF/HSF.shtml).

## Results

We first tested the genotype distribution of the *CDK12* c.1047-2A>G splice site variant in the Hannover-Ufa Breast Cancer Study using genomic DNA samples from breast cancer cases and controls selected for Tatar ancestry where it had been originally described (6). PCR and RFLP analysis identified this variant in 6/155 breast cancer patients (4%) and 12/362 healthy female controls (3%) (Figure 1A; Table 1). The genotype distribution between cases and controls was not significantly different (OR 1.17, 95% CI 0.34–3.45, *p* = 0.79). The median age at diagnosis for *CDK12* c.1047-2A>G carriers was 53.5 years (range 40–61 years) and none had a family history of breast cancer, although one of the six carriers had a first-degree relative with ovarian cancer.

**Table 1:** Distribution of *CDK12* c.1047-2A>G among cases and controls in three ethnic groups from Bashkortostan and Kazakhstan.

**Study population**	**Carriers** **(cases)**	**Carriers** **(controls)**	**OR (95% CI)**	***p***
Tatars (Bashkortostan)	6/155	12/362	1.17 (0.43–3.19)	0.59
Bashkirs (Bashkortostan)	0/96	9/189	n.d.	0.03
Kazakhs (Kazakhstan)	1/131	0/154	n.d.	0.46
Total	7/382	21/705	0.72 (0.30–1.70)^*^	0.45

*Carrier frequencies for CDK12 c.1047-2A>G in patients with breast cancer and in healthy female controls from Bashkortostan and from Kazakhstan*.

*Study populations in Bashkortostan were selected and stratified by ancestry in Tatar and Bashkir subgroups, and genotyping was limited to Kazakh ethnicity in the Kazakhstan study. P-values for single studies were calculated using Fisher's exact test. All p-values are two-sided*.

* *A Mantel-Haenszel odds ratio was derived from a fixed effects meta-analysis with an increment of 0.1 to account for zero fields*.

We then sought to investigate the prevalence of *CDK12* c.1047-2A>G in populations beyond the Tatars and performed a replication study in patients of Bashkir ancestry, also selected from the Hannover-Ufa Breast Cancer Study. In the Bashkir subpopulation, this variant was detected in 0/96 breast cancer cases but in 9/189 healthy female controls (5%). This extended the prevalence of *CDK12* c.1047-2A>G to Bashkirs but the result was suggestive of a protective rather than risk effect for this variant (*p* = 0.03).

Since the *CDK12* c.1047-2A>G variant was present in individuals of both Tatar and Bashkir ancestry, we also genotyped a breast cancer case-control series from Kazakhstan to elucidate its spread toward the Central Asian continent. The variant was present in 1/131 Kazakh breast cancer cases and 0/154 ethnically matched population controls, indicating that it is rare in the Kazakh population.

A fixed-effects meta-analysis of the three substudies yielded a Mantel-Haenszel odds ratio of OR 0.72 (95% CI 0.30–1.70, *p* = 0.45), thus excluding over 2-fold increased risks, with no evidence of heterogeneity between studies (*p*_het_ = 0.23) (Table 1).

We also genotyped genomic DNA samples of 262 unselected patients with epithelial ovarian cancer, drawn from a case series in Bashkortostan that has previously been described (17). We identified four heterozygous carriers (1.5%) of the *CDK12* c.1047-2A>G variant in this group. That carrier frequency was again not significantly different from the 12/362 healthy female controls (3%) in Tatars, but only for one of the four heterozygous ovarian cancer patients a Tatar descent had clearly been documented and population stratification due to mixed ethnicity could not be excluded.

We noticed that the *CDK12* c.1047-2A>G variant site is followed by an alternative splice acceptor site which, if used, would result in the loss of only one codon. The alternative site was predicted to be similarly strong as the reference site with MaxEntScores 6.93 and 6.65, respectively, and while the reference site is completely abolished by the *CDK12* c.1047-2A>G variant, the alternative site would be strengthened in the mutant context (MaxEntScore 8.15) (Figure 1B). When using a second algorithm, Human Splicing Finder, the results were similar with scores of 90 and 87 for the canonical and the alternative NAG on the wildtype allele, and with the alternative NAG score increasing from 87 to 89 with the variant.

We aimed to validate these predictions in patient-derived material and isolated lymphocyte RNA from a patient heterozygous for the *CDK12* c.1047-2A>G variant and from three patients and one healthy female control with wildtype sequence at that position. RT-PCR and sequencing indicated that, in accordance with the MaxEntScan prediction, the alternative site was used in ~30% of wildtype mRNA transcripts in all four individuals with a wildtype splice site whereas this rate increased to some 65% in the heterozygous carrier of the *CDK12* c.1047-2A>G variant (Figure 1C), suggesting that the alternative site is reinforced in the presence of this variant.

## Discussion

Cyclin-dependent kinases (CDKs), a family of proteins harboring a cyclin box, are important regulators driving and coordinating the eukaryotic cell cycle (18), and the development of CDK inhibitors is among the most recent advances in the treatment of breast cancer (19, 20). CDK12 may have a particularly interesting role as a new therapeutic target in oncology as its inhibition acts synthetically lethal with PARP1 inhibition, probably due to the role of CDK12 in homologous recombinational repair (21–23), but it also seems to modulate the antitumor immune response (13). Some 5–15% of many tumors harbor structural changes in *CDK12* which seems to render them susceptible to a tandem duplicator phenotype (10) and, in breast tumors, to an aggressive “BRCAness” phenotype (7). It is thus important to clarify the role of *CDK12* variants for breast cancer risk and treatment.

While truncating *CDK12* germline variants are very rare, the *CDK12* c.1047-2A>G splice variant has recently been reported to occur quite commonly in Tatars where it has been found in some 5% of breast cancer patients and was associated with an about 10-fold increased breast cancer risk (6). This variant, rs138292741, has been recorded in the ExAc database with minor allele frequencies of 0.001 in South Asians and 0.0005 in Europeans, indicating that the Tatar population may harbor a founder effect and therefore might be particularly well suited for association studies regarding this *CDK12* variant. The use of founder populations can strongly increase the power of association studies, though the rarity of mutations can also render these studies prone to false positive results due to population stratification and thus a careful selection of ethnically matched cases and controls is needed. Our study has confirmed the high prevalence of *CDK12* c.1047-2A>G in Tatars but it also occurred in healthy population controls of Tatar or Bashkir ancestry at polymorphic frequencies. The results of our meta-analysis for the three studies in Tatars, Bashkirs and Kazakhs did not indicate an enrichment of carriers in cases and excluded a 2-fold increase in risk of breast cancer. When we include the hypothesis-generating study of Brovkina et al. (6) into the meta-analysis, the combined OR 1.37 (95% CI 0.70; 2.69) still does not confirm a role as susceptibility factor and indicates that any risk conferred by the *CDK12* variant is very unlikely to be higher than 3-fold.

The *CDK12* c.1047-2A>G may not be representative for a typical loss-of-function mutation in *CDK12*. Although it affects a conserved splice acceptor site and a skipping of the downstream exon would cause a loss of 295 amino acids, this site is followed by an alternative splice acceptor site the use of which would result in the loss of only one codon, a so-called NAGNAG sequence context (24). The alternative site was predicted to be similarly strong as the reference site with MaxEntScores 6.93 and 6.65, respectively, and while the reference site is completely abolished by the *CDK12* c.1047-2A>G variant, the alternative site is strengthened in the mutant context (MaxEntScore 8.15). This *in silico* analysis is highly predictive (25) and indicates a potentially mild effect of the *CDK12* c.1047-2A>G variant. A similar result was predicted using HSF as a second algorithm. Finally, our RT-PCR analyses for a heterozygous carrier indicated an increase in the usage of the alternative downstream acceptor site that was fully consistent with the predicted effect for the variant allele. Although it is still possible that other factors can modulate this splicing event, the most parsimonious interpretation of these data is that the *CDK12* c.1047-2A>G variant reinforces the downstream acceptor site. Alternative splicing at NAGNAG acceptors is widespread and contributes to proteome plasticity (24). Such NAGNAG acceptor sites can amelioriate or bypass the phenotype of a mutation (26), but some can also act in a tissue-specific manner (27). In the present study, we had no possibility to determine the ratio of the protein isoforms and assess their stability and function in breast epithelial tissue. If *CDK12* c.1047-2A>G has functional consequences, even due to the loss of a single amino acid Arg349 outside the catalytic domain, our results would still be compatible with a minor risk in the range of low-penetrance variants many of which have been detected through genome-wide association studies worldwide (16, 28). Very large study sizes, perhaps assisted by segregation analyses from multiple-case families, would be required to detect such modest effects.

In summary, our study has confirmed a high prevalence of a *CDK12* c.1047-2A>G splice site variant in the Tatar as well as in the Bashkir population but provides evidence for alternative splicing as a likely attenuating mechanism for this variant. While minor risks for breast cancer cannot be excluded, our case-control analyses indicate that *CDK12* c.1047-2A>G does not represent a clinically actionable mutation.

## Data Availability

All datasets generated for this study are included in the manuscript and/or the supplementary files.

## Ethics Statement

The experiments in the present study comply with the current laws of the country in which they were performed. This study was carried out in accordance with the recommendations of the Ethics Commission at Hannover Medical School with written informed consent from all subjects. All subjects gave written informed consent in accordance with the Declaration of Helsinki. The protocol was approved by the Ethics Commission at Hannover Medical School.

## Author Contributions

TD, NT, and EK: designed the study. NB, PS, and YV: performed the genotyping and cDNA analyses. ND, NT, TY, ZE, EM, DP, MB, and EK: provided material and data. NB and TD: analyzed the data. TD: prepared the draft and all authors approved the manuscript.

### Conflict of Interest Statement

The authors declare that the research was conducted in the absence of any commercial or financial relationships that could be construed as a potential conflict of interest.
